# Cost-effectiveness analysis of cochlear dose reduction by proton beam therapy for medulloblastoma in childhood

**DOI:** 10.1093/jrr/rrt112

**Published:** 2013-11-01

**Authors:** Emi Hirano, Hiroshi Fuji, Tsuyoshi Onoe, Vinay Kumar, Hiroki Shirato, Koichi Kawabuchi

**Affiliations:** 1 Tokyo Medical and Dental University, Faculty of Dentistry, School of Dentistry, 1-5-45 Yushima, Bunkyou-ku, Tokyo, 113-8510 Japan; 2 Shizuoka Cancer Center, 1007 Shiomonagakubo, Nagaizumi-cho, Sunto-gun, Shizuoka-pref. 411-8777, Japan; 3 All India Institute of Medical Sciences, New Delhi, 110029, India; 4 Hokkaido University Graduate School of Medicine, Kita 15, Nishi 7, Kita-ku, Sapporo, Hokkaido, 060-0808, Japan

**Keywords:** medulloblastoma, proton therapy, cost effectiveness, hearing loss

## Abstract

Background: The aim of this study is to evaluate the cost-effectiveness of proton beam therapy with cochlear dose reduction compared with conventional X-ray radiotherapy for medulloblastoma in childhood. Methods: We developed a Markov model to describe health states of 6-year-old children with medulloblastoma after treatment with proton or X-ray radiotherapy. The risks of hearing loss were calculated on cochlear dose for each treatment. Three types of health-related quality of life (HRQOL) of EQ-5D, HUI3 and SF-6D were used for estimation of quality-adjusted life years (QALYs). The incremental cost-effectiveness ratio (ICER) for proton beam therapy compared with X-ray radiotherapy was calculated for each HRQOL. Sensitivity analyses were performed to model uncertainty in these parameters. Results: The ICER for EQ-5D, HUI3 and SF-6D were $21 716/QALY, $11 773/QALY, and $20 150/QALY, respectively. One-way sensitivity analyses found that the results were sensitive to discount rate, the risk of hearing loss after proton therapy, and costs of proton irradiation. Cost-effectiveness acceptability curve analysis revealed a 99% probability of proton therapy being cost effective at a societal willingness-to-pay value. Conclusions: Proton beam therapy with cochlear dose reduction improves health outcomes at a cost that is within the acceptable cost-effectiveness range from the payer's standpoint.

## INTRODUCTION

The medulloblastoma is a common embryonal tumor occurring in the cerebella, and a commonly occurring childhood brain tumor. According to national statistics of brain tumors in Japan, medulloblastomas comprise 1.1% of all brain tumors and 11.9% of childhood brain tumors [>^1^]. It is estimated that ∼ 80 patients are newly diagnosed with medulloblastomas annually [2, 3].

Medulloblastomas were known to lead to death about 50 years ago, but in recent decades multidisciplinary therapy combining surgery, radiotherapy and chemotherapy has been established, and treatment results for medulloblastomas have greatly improved [4]. Currently, 5-year survival rates are 85% in an average-risk group [5] and 70% in high-risk groups [5] treated with chemotherapies comprised of vincristine, cisplatin (CDDP) and cyclophosphamide after craniospinal irradiation (CSI) followed by tumor bed irradiation. However, along with the improvements in survival rates of children suffering from medulloblastomas, complications arising in the later stages, which long-term survivors suffer all through life, present problems. Hearing loss caused by using CDDP and radiotherapies is the most frequently experienced late stage complication of medulloblastoma treatment, with the exception of central nervous system disorders [6–8]. Hearing loss severely affects the development of child patient communication abilities and social skills, leading to a poorer quality of life (QOL) [9].

Hearing loss caused by using CDDP and radiotherapies is known to occur even with low doses of irradiation, and is correlated with the administered cochlear doses [10]. Thus, many investigations have been conducted to alleviate hearing losses by using state-of-the-art radiotherapies. Specifically, proton beam therapy is considered to lower the dose that organs in the vicinity of targets are exposed to and is considered to be the most effective radiotherapy for treating childhood tumors. The advantages arise because proton beams display a characteristic known as the Spread-Out Bragg Peak, a shape of the dose distribution that enables an accurate fit to the target shape, even when only few beams from different directions are used.

One notable disadvantage of proton beam therapy is cost. It requires a large-scale facility that is expensive to construct and operate, resulting in higher medical expenses than with conventional X-ray therapy. There is a study by Lundkvist *et al.* [11] on the cost effectiveness of proton beam therapy for medulloblastomas, conducted before proton beam CSI became common, which reported that proton beam therapy is cost-saving rather than cost-effective, when compared with conventional X-ray therapy. However, the model of Lundkvist *et al.* assumed the incidence of adverse events due to proton beam therapy to be 0.12 times those due to X-ray therapy, but the model was not based on the dose to organs in actual proton beam therapies. Therefore, any conclusion that proton beams will alleviate adverse events has not been fully validated [12]. During the last decade the number of facilities for proton beam therapies has rapidly increased, and this kind of therapy for medulloblastomas has become commonly employed. This situation enables a comparison of cochlear doses using proton beams with those using X-ray therapies, by analyzing data from actual cases. The increase in the number of facilities for proton beam therapies has also made it necessary to consider appropriate allocation policies for such facilities and to clearly evaluate levels of medical fees, and there is a need for more relevant analyses of cost-effectiveness.

This study defines the cochlear doses in proton beam and in X-ray therapies by analyzing data from actual cases, and estimates the risks of hearing loss from these therapies. The cost-effectiveness of proton beam therapies compared with that of conventional X-ray therapies is examined based on a risk analysis of hearing loss.

## MATERIAL AND METHODS

### Decision model

**Figure 1.**
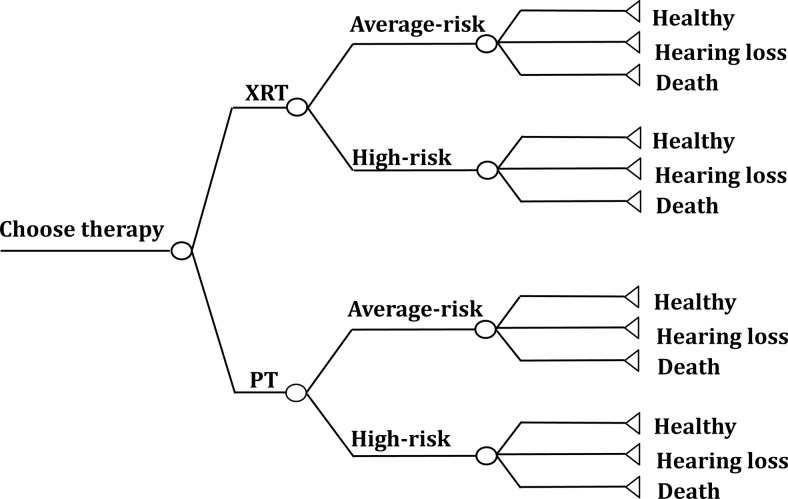
Markov model. The model used to compare X-ray radiotherapy with proton beam therapy in the treatment of a patient with medulloblastoma. XRT = X-ray radiotherapy, PT = proton radiotherapy.

A cost-utility analysis was conducted for the annual incidence of the Japan cohort of child patients with medulloblastomas, using a decision tree and the Markov model. In this study the cohort of six-year old patients who underwent therapies for medulloblastomas was classified into two groups: a group treated with conventional X-ray therapies (XRT group) and a group treated with proton beam therapies (PT group) (Fig. 1). It was assumed that both groups had undergone standard treatments other than the radiotherapy, and based on the above, the disease models were estimated. The model simulations were conducted from the time when each child patient had undergone a therapy till death or till reaching 100 years of age [11].

### Model assumptions and data

This study was approved by the Institutional Review Board of the Shizuoka Cancer Center, and informed consent was waived because of the retrospective nature of the study. Table 1 shows the variables used in creating the Markov model. Currently, this model is used as the standard therapy for medulloblastomas. The therapies were performed in accordance with the protocol of St Jude Medulloblastoma-96 (SJMB-96). With this protocol, CSI with the proton beam is conducted after excision of as much of the tumor as possible, and booster irradiation to the tumor bed is conducted followed by four courses of chemotherapy including CDDP.

**Table 1.** Probabilities, utility values and costs used in study

Event	Baseline value	Range studied	Reference
Probabilities			
5-year OS			
Average-risk	85.00%	75–94%	5
High-risk	70.00%	54–84%	5
Risk of Grade 3–4 hearing loss			
XRT: average-risk	39.00%	36.99–41.00%	6, 15
XRT: high-risk	47.11%	44.55–49.67%	6, 15
PT: average-risk	15.55%	4.97–26.12%	6, 15
PT: high-risk	26.53%	18.36–34.71%	6, 15
Rate of high-risk group	30%	25–35%	13
Utility values			
EQ-5D	0.807	0.784–0.830	16
HUI3	0.644	0.626–0.663	16
SF-6D	0.792	0.779–0.804	16
Costs			
Radiation cost			
X-ray radiation	$3 082.20	$2 311.7–$3 852.8	
Proton radiation	$26 943.90	$20 207.9–$33 679.9	
Hearing test	$65.4		
Hearing aid fitting test	$121.5		
Hearing aid	$2 086.9	$1 565.2–$2,608.6	17

**Figure 2.**
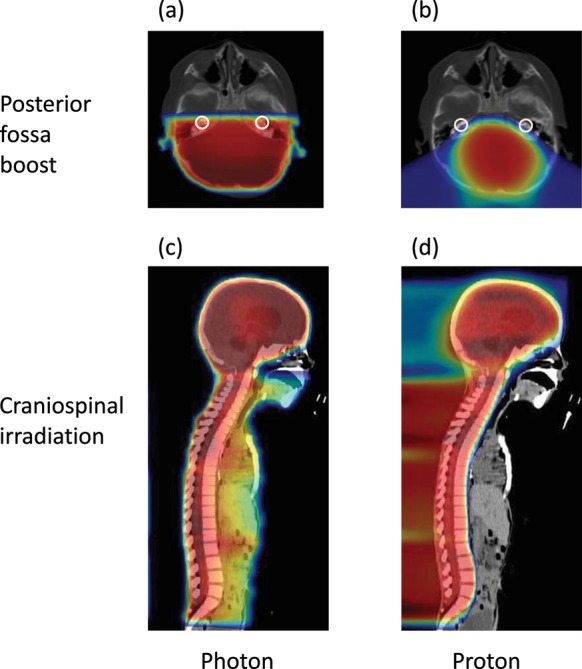
Treatment planning data of a representative case with medulloblastoma. Dose distribution in the transaxial plane of the posterior fossa for boost irradiation to the tumor bed with X-ray (**a**) and proton (**b**) beams. Dose distribution of sagittal plane for craniospinal irradiation with X-ray (**c**) and proton (**d**) beams. The cochleae are indicaed by white circles.

With medulloblastomas, since the choice of therapy depends on a classification into an average-risk group (M0 and residual diseases < 1.5 cm^2^) or a high-risk group (M1–3 or residual diseases ≥ 1.5cm^2^) [5], 70% and 30% of the XRT group and PT group were assumed to belong to the average-risk and the high-risk groups, respectively [13]. The same doses as prescribed for the X-ray therapies were used in the proton beam therapies. For CSI with both X-rays and proton beams, 23.4 Gy and 30.6 Gy are administered to the average-risk and high-risk groups, respectively. For additional irradiation to the tumor bed, 30.6 Gy and 23.4 Gy are administered to the average-risk and high-risk groups, respectively. With this model, the cochlear doses for the proton beam therapy were calculated using the data from treatment plans for eight patients who underwent proton beam therapy. Further, for the same patients, cochlear doses for X-ray therapy were calculated by conducting simulations of treatment plans. Figure 2 shows dose distributions of the proton beam therapy and X-ray therapy for the patients used in the development of the model. A dose identical to that intended for the target is irradiated onto the cochlea, both in the X-ray CSI and in the proton beam CSI. However, for the irradiation to the tumor bed, irradiation with the proton beam shows higher conformity than that of the X-rays, and the cochlear dose is reduced [14]. The estimated cochlear doses for X-ray treatment in the average-risk and the high-risk groups were 46.4 Gy and 50.0 Gy, respectively; those for proton beam treatment were 29.3 Gy and 39.6 Gy, respectively. The development of hearing loss due to the treatment of cerebral tumors coupled with CDDP is correlated with the cochlear irradiation dose and to CDDP doses [6, 10].

With this protocol, the total accumulated dose of CDDP administered is ∼ 300 mg. Studies by Schell *et al.* [6] and Huang *et al.* [15] reported a relation between Grade 3–4 hearing loss incidence (HL) and the dose irradiated to the cochlea when similar doses of CDDP were used. The logistic regression equations that show the relations between cochlear radiation dose and hearing loss appearance were created based on these two studies, and the curve of the dose-effect relation was obtained from the average of the two equations.

In this study, the time from the onset of treatment to the incidence of hearing loss was defined as one year, and it was assumed to reach a plateau from the third year onward from previous studies [10, 15]. There is no difference in the treatment effect for the XRT and PT groups because the target dose for both groups is the same. The death rate of the XRT and PT groups was assumed to be the same as there is no possibility of the occurrence of fatal complications, although the XRT group had higher doses irradiated on organs in the vicinity of the tumor bed. From previous studies, the 5-year survival rates were defined as 85% (95% CI: 75–94) for the average-risk group and 70% (95% CI: 54–84) for the high-risk group, and the survival rate past 5 years was defined as a plateau [5]. However, the death rate from 6 years onward was calculated by including causes of death other than medulloblastomas, using the weighted average efficiency of the death rates based on gender and age, based on the data in Life Table No. 21 (Complete Life Table).

### Utility values

Hearing loss due to medulloblastoma treatment is irreversible, and patients who develop hearing loss need to use hearing aids for the rest of their lives. Barton *et al.* [16] compared utility values before and after the use of hearing aids by using three Health Related Quality of Life indexes (HRQOL): EQ-5D, HUI3 and SF-6D. Here, the utility values of EQ-5D, HUI3, and SF-6D after using hearing aids are 0.807, 0.644 and 0.792, respectively (Table 1). Although the value of HUI3 is lower than those of EQ-5D and SF-6D, this may be due to the characteristics of the items questioned. It is noteworthy that HUI3 is comprised of eight attributes, and of these the items related to Hearing and Cognition yield particularly low values. However, as HUI3 may still be more sensitive to hearing loss than EQ-5D and SF-6D, this study analyzed the results with the three utility indexes separately.

### Costs

The costs to be analyzed are limited to direct medical costs for the radiotherapy and direct medical costs related to hearing loss, and exclude indirect costs such as work loss and direct non-medical costs (including transportation costs), because the analyses were conducted from the perspective of the healthcare payers. Table 1 shows the amounts of the costs. The medical costs, including that of X-ray irradiation, were derived from the Table of Medical Service Fees in 2012 in Japan. However, since proton beam therapy is not listed in the medical fee table, this study used the median of the treatment fees of medical institutions in Japan for this kind of treatment.

Further, patients with Grade 3–4 hearing loss need hearing aids. Therefore, the medical costs after the onset of hearing loss include expenses for tests at the time of the onset of hearing loss and the resultant costs of purchasing hearing aids after the onset of the hearing loss. The expenditure on hearing assessment here include expenses for a hearing test and a hearing aid fitting test after the onset of the hearing loss, and annual hearing tests for next two years after that. This study adopted the hearing aids cost of $2087 (JPY223 300) reported by Kataoka *et al.*, and the durability as 5 years [17].

The effect analysis, the quality adjusted life years (QALY) were used as an index in view of the importance of life-long QOL of patients with medulloblastoma. Calculating an incremental cost-effectiveness ratio (ICER) as a final index of the cost-utility analysis, the economic efficiency was evaluated based on a societal willingness to pay value (WTP). This study used $46 729/QALY (JPY 5 million/QALY) as the threshold of standard for the economic evaluation [18]. The discount rate that computes the cost and effect in the present values was set to 3% [18].

### Sensitivity analyses

This study conducted a one-way sensitivity analysis and a probabilistic sensitivity analysis with the range shown in Table 1. Assuming the variability at a 95% confidence interval to be ±25%, the discount rates were assumed as 0–7%. A probabilistic sensitivity analysis was conducted using Monte Carlo simulations, where a trial with all the variables fluctuating simultaneously under the probability distributions was performed 10 000 times. The probability distribution of each variable was deemed to be a triangular distribution. Further, cost-effectiveness acceptability curves (CEAC) were developed based on the result of the Monte Carlo simulations, where CEAS describes the probabilities that meet specific conditions related to continuous WTP values as well as to a specific WTP value.

## RESULTS

### Base case

For the base-case analysis, the ICERs for EQ-5D, HUI3 and SF-6D were $21 716/QALY (JPY 2 323 602/QALY), $11 773/QALY (JPY 1 259 706/QALY) and $20 150/QALY (JPY 2 156 034/QALY), respectively (Table 2). These ICER utility values were all lower than the $46 729/QALY (JPY 5 million /QALY), which is considered a threshold value for including a treatment among standard therapies in Japan. The ICER of HUI3 is lower than those of EQ-5D and SF-6D.

**Table 2.** Results of Markov model analysis by utility: per patient.

	Cost	QALY	ΔCost	ΔQALY	ICER($/QALY)
EQ-5D					
proton therapy	$28 937.00	23.44	21 396	0.98	21 716
X-ray therapy	$7 541.00	22.46	…		
HUI3					
proton therapy	…	22.78	…	1.82	11 773
X-ray therapy	…	20.96	…		
SF-6D					
proton therapy	…	23.38	…	1.06	20 150
X-ray therapy	…	22.32	…		

QALY = quality-adjusted life years, ICER = incremental cost-effectiveness ratio.

### Sensitivity analyses

**Figure 3.**
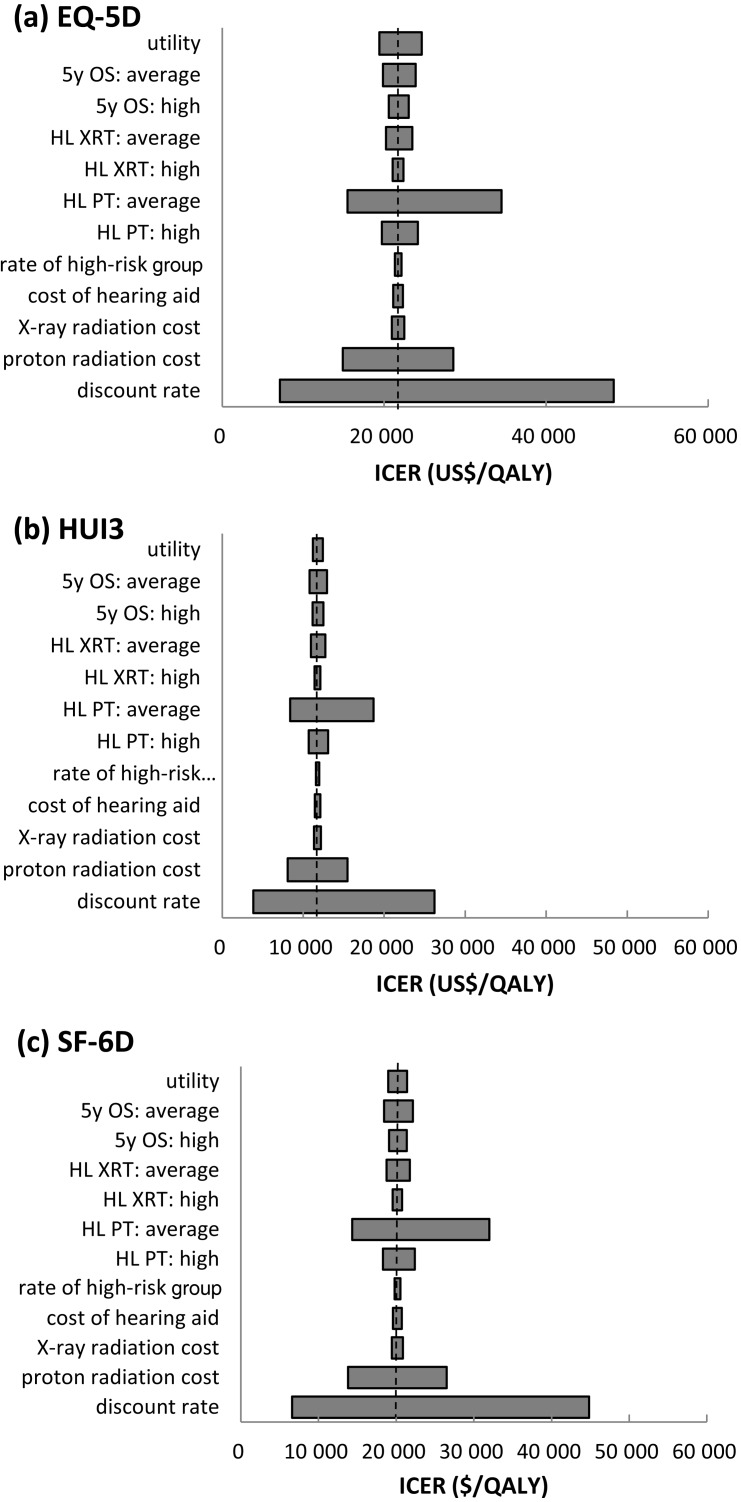
One-way sensitivity analyses by EQ-5D (**a**), HUI3 (**b**) and SF-6D (**c**). 5y OS = 5-year overall survival, HL = hearing loss, XRT = X-ray radiotherapy, PT = proton beam therapy, and ICER = incremental cost-effectiveness ratio.

**Figure 4.**
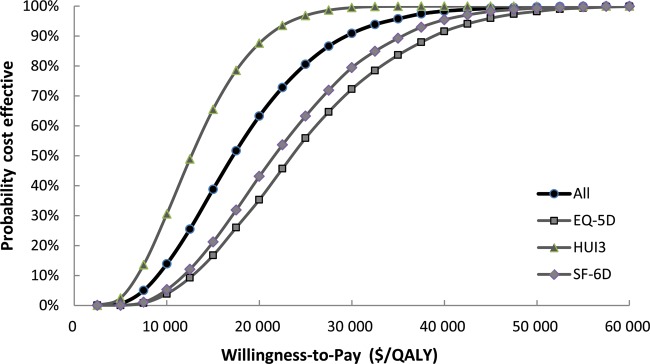
Incremental cost-effectiveness acceptability curve for EQ-5D, HUI3, SF-6D and all three integrated.

The results of the one-way sensitivity analysis for each of the utility indexes are shown in Fig. 3. The variable with the broadest range is the discount rate, followed by hearing loss incidence (HL) and treatment costs for the proton irradiation of the average-risk group. The results of the probabilistic sensitivity analysis by the Monte Carlo simulations are shown in Fig. 4. Most trials yielded values lower than $46 729/QALY (JPY 5 million/QALY), which is considered the threshold value. The cost-effectiveness acceptability curve obtained for the accuracy showed that the probability of WTP being lower than $46 729/QALY (JPY 5 million/QALY) is 99.51%. The WTPs for EQ-5D, HUI3 and SF-6D are 96.95, 100 and 98.72%, respectively. Consequently, it is suggested that the proton beam therapy has an excellent probability of being cost-effective.

## DISCUSSION

This study evaluates the cost-effectiveness of proton beam therapy for medulloblastomas in children by comparing it with that of conventional X-ray radiotherapy. The analysis showed that ICER values for EQ-5D, HUI3 and SF-6D were $21 716/QALY (JPY 2 323 602/QALY), $11 773/QALY (JPY 1 259 706/QALY) and $20 150/QALY (JPY 2 156 034/QALY), respectively. As described above, the HUI3 can be considered more sensitive to hearing loss than EQ-5D and SF-6D, and further, the ICER of HUI3 is lower than those of the other two. Additionally, for all of the utility indexes the ICERs were lower than the $46 729/QALY (JPY 5 million/QALY), which is considered the WTP in Japan, and the robustness of this conclusion was shown by a sensitivity analysis. Therefore, the results of the evaluation here suggest that proton beam therapy is a more cost-effective therapy than conventional X-ray therapy.

The results of the one-way sensitivity analysis show that the variable with the third largest ICER range of variability is the treatment cost of proton beam therapy. This suggests that if a facility for proton beam therapy could be scaled down in size, the construction of proton beam facilities would involve much lower costs than at present, and this would make proton beam therapy even more cost-effective.

The cost-effectiveness analysis in the study presented here focuses on reductions in the hearing loss accompanying the treatments. If proton beam therapy is used, the doses irradiated onto a range of organs will be reduced; it has been reported that the doses irradiated onto the cerebrum, hypophysis, parotid gland, thyroid gland, lungs, heart and liver can be reduced [19, 20]. It is clear that reducing doses in the medulloblastoma treatment has the most significant clinical effect on hearing loss of all the disorders in organs arising from the therapy. This is because hearing loss due to the irradiation on the cochlea occurs significantly often and is severe, and a correlation between the dose and the risk of hearing loss associated with medulloblastoma treatment has been established [6, 15].

By selecting the cochlea as the organ to be evaluated in terms of benefit from proton beam therapy, we have avoided overestimation of the benefit of the treatment by the approximation of risk estimates, without the validation of actual dose reduction and the dose–response relationship. Further, the cost-effectiveness analyses of hearing loss can yield measurable results, as the effect of the medulloblastoma treatment can be expressed as a utility value.

Although significant disorders occur in organs other than the cochlea, where the irradiation dose is able to be reduced by using proton beam irradiation, the utility values for patients with combined toxicities will not be available until long-term observation of the patients after proton beam therapy has been performed. The amount necessary to compensate for the hearing loss, such as the price of hearing aids, is effective in yielding measurable results.

With intensity-modulated radiation therapy (IMRT), which improves conformity of dose distribution effects, as with proton beam irradiation, improvements in the cost-effectiveness can be expected by using the hearing loss findings as indexes. With IMRT, it is possible to reduce doses irradiated to peripheral, unaffected tissue by combining radiation beams of different angles. In medulloblastoma treatment, IMRT has been shown to lower the risk of hearing loss by reducing the cochlear doses [15].

However, concerns about IMRT have been expressed, as some organs are exposed to higher doses of radiation than with the generally administered X-ray therapy. A further concern with IMRT is the occurrence of cancers induced by the radiation exposure when the whole body of the patient is exposed to multiple low dose beams from many directions, resulting in overall increases in the doses, especially for children [14]. Thus, it is premature to discuss the cost-effectiveness focusing on hearing loss as an index if there are increased risks of other organs being damaged while reducing the risk of hearing loss. Proton beam therapy can reduce doses to organs other than the cochlea and presents no possibility of increasing the risk of damage to other organs; therefore, eliminating the risk of lowered damage to another organ is an advantage of the proton beam therapy.

The study reported here was not able to the implement a utility value for the society of the study model. A few clinical studies have evaluated the QOL necessary for a calculation of the QALY after radiotherapy. Further, the number of studies focusing on children or Japanese people is very small. Therefore, we implemented established HRQOLs for adults in western countries for this analysis. Although identical results were obtained by using values varying widely from 0.644 to 0.807, validation for the values for Japanese children and adults is required for specifying the precise ICER of proton beam therapy in the Japanese health care system.

As a pioneering study of the cost-effectiveness of proton beam therapies in Japan, this study, focusing on medulloblastomas in children, is expected to contribute to minimizing the influence of limitations related to parameters such as incidence probability, QALY and cost. In this study, the cost-effectiveness and societal affordability of proton beam therapy for medulloblastomas in children has been suggested; however, cost-effectiveness analyses for other diseases including lung cancer, prostate cancer and breast cancer still need to be addressed. Therefore, further clinical research related to proton beam therapy and examining its economic effectiveness and medical utility is desirable.

## FUNDING

This study was supported in part by grants from The Funding Program for World-Leading Innovative R&D on Science and Technology (FIRST program) and by the National Cancer Center Research and Development Fund (23-A-21).
